# PathoNet introduced as a deep neural network backend for evaluation of Ki-67 and tumor-infiltrating lymphocytes in breast cancer

**DOI:** 10.1038/s41598-021-86912-w

**Published:** 2021-04-19

**Authors:** Farzin Negahbani, Rasool Sabzi, Bita Pakniyat Jahromi, Dena Firouzabadi, Fateme Movahedi, Mahsa Kohandel Shirazi, Shayan Majidi, Amirreza Dehghanian

**Affiliations:** 1 Department of Computer Science and Engineering, Shiraz University, Shiraz, Iran; 2 Department of Pathology, Shiraz University of Medical science, Shiraz, Iran; 3 Student Research Committee, Shiraz University of Medical Science, Shiraz, Iran; 4 Department of Clinical Pharmacy, Shiraz University of Medical Sciences, Shiraz, Iran; 5 Molecular Pathology and Cytogenetics Division, Department of Pathology, Shiraz University of Medical Sciences, Shiraz, Iran; 6 Trauma Research Center, Shiraz University of Medical Sciences, Shiraz, Iran; 7 Present Address: Department of Computer Science and Engineering, Koc University, Istanbul, Turkey

**Keywords:** Cancer, Molecular medicine, Oncology, Mathematics and computing

## Abstract

The nuclear protein Ki-67 and Tumor infiltrating lymphocytes (TILs) have been introduced as prognostic factors in predicting both tumor progression and probable response to chemotherapy. The value of Ki-67 index and TILs in approach to heterogeneous tumors such as Breast cancer (BC) that is the most common cancer in women worldwide, has been highlighted in literature. Considering that estimation of both factors are dependent on professional pathologists’ observation and inter-individual variations may also exist, automated methods using machine learning, specifically approaches based on deep learning, have attracted attention. Yet, deep learning methods need considerable annotated data. In the absence of publicly available benchmarks for BC Ki-67 cell detection and further annotated classification of cells, In this study we propose SHIDC-BC-Ki-67 as a dataset for the aforementioned purpose. We also introduce a novel pipeline and backend, for estimation of Ki-67 expression and simultaneous determination of intratumoral TILs score in breast cancer cells. Further, we show that despite the challenges that our proposed model has encountered, our proposed backend, PathoNet, outperforms the state of the art methods proposed to date with regard to harmonic mean measure acquired. Dataset is publicly available in http://shiraz-hidc.com and all experiment codes are published in https://github.com/SHIDCenter/PathoNet.

## Introduction

The nuclear protein Ki-67 was first detected in Hodgkin lymphoma cell line and introduced as a proliferative marker^1^. It was further confirmed that the monoclonal antibody Ki-67 is present during all cell cycle phases except for the G0^2^. However variation in its extent of expression has been reported during different phases, as for the G1 accounting for the lowest^3^. Knowing that excessive cellular proliferation correlates with progression of malignancy, precise estimation of this protein marker can benefit physicians in identifying high-grade tumors and can also convey prognostic value in approach to tumor management^4–6^. Also tumoral cells may suppress the body’s immune mechanisms, however tumor infiltrating lymphocytes (TILs), introduced as an immune component against tumor progression, have been found beneficial in improving outcome of breast cancer patients^7–10^. On the other hand, heterogeneity of breast cancer complicates approach to its treatment.Accurate quantitative determination of markers such as Ki-67 and TILs can simplify this approach to some extent. The established method for Ki-67 detection is Immunohistochemical (IHC) analysis using MIB-1 or SP6 as monoclonal antibodies used in the staining process performed on paraffin embedded tissue^11,12^. TILs scoring is also evaluated using the same tissue blocks using recommendations by the international TILs working group^8^. Considering that both markers’ scoring is based on an expert pathologist’s decision, inter-observer result variations are inevitable. To increase the accuracy of estimation, it has been suggested to count all tumor cells from different fields of a breast tissue section. If impossible to do so, at least 500–1000 cells in the representative areas of the whole section are recommended to be counted by the pathologist^12^. This, in turn, can be very time consuming for large numbers of samples The mentioned limitations prompt the need for an exact continuous calculation of both markers, which could be assessed by means of Artificial intelligence (AI).

AI has significantly improved the speed and precision of clinical diagnosis and is becoming an inseparable part of different aspects of medicine^13^. Before introducing deep learning, conventional AI algorithms were commonly used; however, designing a generalized and robust method requires field experts to extract handcrafted features. By the advent of deep neural networks, having the facility of automatically learning the best features from the input data, this issue has almost been resolved. In addition, if these algorithms are developed and trained with diverse and adequate data, they are generalizable and robust. Convolutional neural network (CNN) is a class of deep neural networks widely used in different areas such as Robotics, Bioinformatics, Computer Vision, etc. and have shown to be highly efficacious specifically, in image processing^14–16^.

Although Neocognitron introduced by Fukushima et al. in 1979 and later CNNs were introduced by LeCun et al. in 1989 for handwritten digit classification, CNNs failed to be much successful due to computational barriers and lack of sufficient data. In 2012, improvement of CNN’s computational capability was observed in introduction of AlexNet^17^, which won the ImageNet competition using a CNN. To overcome the shortcomings of manual assessment of Ki-67 and TILs and yet to take advantage of the probable favorable role of both markers in approach to breast cancer, in this experimental study we have designed and suggested the use of AI assisted methods with emphasis on CNN for the more accurate detection of tumoral cells along with Ki-67 and TILs. Different aspects of this study can be summarized into four categories. First, a dataset with detection and classification annotation has been introduced that provides a benchmark for Ki-67 stained cell detection, classification, proliferation index and tumor infilterating lymphocytes (TILs) estimation. Second, we suggest a novel pipeline that can achieve cell detection and classification and further examined the proposed pipeline on our benchmark. Third, we recommend a deep network, named PathoNet, that outperforms the state of the art backends with the proposed pipeline in Ki-67 immunopositive, immunonegative, and lymphocyte detection and classification. Lastly, we introduce a residual inception module that provides higher accuracy without causing vanishing gradient or overfitting issues.

## Literature review

Data regarding detection and estimation of Ki-67 by means of deep learning and conventional machine learning algorithms are present in the literature. Some conventional methods have been suggested in this regard; A study on neuroendocrine tumors (NET) presented a framework for Ki-67 assessment of NET samples that can differentiate tumoral from non-tumoral cells (such as lymphocytes) and has furthermore classified immunopositive and immunonegative tumor cells to achieve automatic Ki-67 scoring^18^. For the tumor biopsies of meningiomas and oligodendrogliomas based on immunohistochemical (IHC) Ki-67 stained images, Swiderska et al., introduced a combination of morphological methods, texture analysis, classification, and thresholding^19^. Shi et al. carried out a study based on morphological methods to address color distribution inconsistency of different cell types in the IHC Ki-67 staining of nasopharyngeal carcinoma images. They suggested classifying image pixels using local pixel correlations taken from specific color spaces^20^. Geread et al. proposed a robust unsupervised method for discriminating between brown and blue colors^21^.

Despite improvements, conventional methods not only lack generalization and accuracy compared to direct interpretation by pathologists, they are also complex to develop because of having handcrafted features. As for Deep Learning methods, different aspects including image classification, cell detection, nuclei detection, and Ki-67 estimation in histopathological images have been studied and reported. Xu et al. suggested using deep learning features in multiple instance learning (MIL) framework for colon cancer classification^22^. Weidi et al. proposed a cell spatial density map using CNNs to overcome interference of cell clumping or overlapping while performing task of automated cell counting and detection^23^.

On the other hand, Cohen et al. suggested redundant counting instead of proposing a density map. Moreover, they introduced a network derived from inception networks called Count-ception for cell counting^24^. Spanhol et al. compared conventional methods with deep features in breast cancer evaluation^25^. In another study on breast cancer Ki-67 scoring by Saha et al., decision layers were used in detecting hotspots using gamma mixture model assisted deep learning^26^. Zhang et al. used CNN to classify images as benign or malignant and a single shot multibox detector as an object detector to assess Ki-67 proliferation score in breast biopsies^27^. Sornapudi et al. extracted localized features by taking advantage of superpixels generated using clustering algorithms and thereafter applied a CNN for nuclei detection on extracted features^28^. Due to the restriction of manually labeled Ki-67 datasets, Jiang et al. proposed a new model consisting of residual modules and Squeeze-and-Excitation(SE) block named small SE-ResNet, which has fewer parameters in order to prevent the model from over-fitting. Similar classification accuracy was reported for SE-ResNet compared to the ResNet in classifying samples into benign and malignant^29^. Liu et al. addressed cell counting problem as a regression problem by producing cell density map in a preprocessing step and further utilized a stacked deep CNN model for counting^30^.

Available datasets in form of publicly presented benchmarks can be divided into the benign-malignant classification and cell counting categories. For benign-malignant image classification, Spanhol et al. introduced BreakHis, which consists of breast cancer histopathological images obtained from partial mastectomy specimens from 82 patients with four different magnifications^31^. Diverse cell counting and nuclei detection datasets such as synthetically generated VGG-CellS^32^, real samples of human bone marrow by Kainz et al.^33^, Modified Bone Marrow (MBM) and human subcutaneous adipose tissue (ADI) datasets by Cohen et al.^24^, and Dublin Cell Counting (DCC) proposed by Marsden et al.^34^ are all of the many examples of datasets presented. However, none of the mentioned benchmarks provide facilities for both cell detection and classification. To the best of our knowledge, SHIDC-B-Ki-67 is the first benchmark introducing IHC marked breast cancer specimens that has cell annotations in three different classes of immunopositive, immuno negative, and tumor infiltrating lymphocytes.

## Dataset

The critical role of providing the accurate data for developing deep learning models is evident to experts in this field. In this study the unavailability of a comprehensive Ki-67 marked dataset, lead us to gathering SHIDC-B-Ki-67 by using numerous and various data labeled by expert pathologists. This dataset contains microscopic tru-cut biopsy images of malignant breast tumors exclusively of the invasive ductal carcinoma type (Table 1). Images were taken from biopsy specimens gathered during a clinical study from 2017 to 2020. SHIDC-B-Ki-67 contains 1656 training and 701 test data. Detailed statistics of the annotated cells have been elaborated in Table 2. All patients who participated in this study were patients with a pathologically confirmed diagnosis of breast cancer whose breast tru-cut biopsies were taken at Shiraz University of Medical Sciences’ affiliated hospitals’ pathology Laboratories in Shiraz, Iran. Shiraz University of Medical Sciences institutional review and ethical board committee approved the study (ethics approval ID: IR.SUMS.REC.1399.756) and written informed consent was gathered from all patients willing to take part in the study. All procedures including slide preparation, staining and image acquirement’s were performed according to institutional policies and regulations. Moreover, all the data were anonymized.

**Table 1:** Tumor characteristics of breast cancer patients enrolled for sample collection.

Pt	S	A (y)	Tumor laterality	Tumor type	Tumor size (cm)	Nottingham grade	Node involvement	TNM status	Tumor stage	C Ki-67 index (%)	C TILs score (%)
1	F	54	RIGHT	IDC	3	II/III	YES	T2N2M0	IIIA	10.66	9.58
2	F	41	LEFT	IDC	2	II/III	NO	T1N0M0	IA	19.98	1.91
3	F	55	RIGHT	IDC	1.2	I/III	NO	T1N0M0	IA	11.17	0.53
4	F	59	RIGHT	IDC	2	I/III	NO	T1N0M0	IA	29.39	0.75
5	F	57	LEFT	IDC	3.5	I/III	NO	T2N0M0	IIA	28.82	2.52
6	F	30	RIGHT	IDC	4	II/III	NO	T2N0M0	IIA	75.06	10.98
7	F	44	RIGHT	IDC	2	III/III	NO	T1N0M0	IA	78.81	0.63
8	F	29	LEFT	IDC	2	III/III	NO	T1N0M0	IA	35.24	1.76
9	F	50	RIGHT	IDC	2.3	II/III	NO	T2N0M0	IIA	22.97	3.7
10	F	29	LEFT	IDC	2	III/III	YES	T1N1M0	IIA	52.51	5.81
11	F	43	RIGHT	IDC	3.6	I/III	YES	T2N1M0	IIB	56.98	0.71
12	F	45	LEFT	IDC	1.8	I/III	YES	T1N2M0	IIIA	22.67	0.72
13	F	42	LEFT	IDC	2	III/III	YES	T2N2M0	IIIA	23.14	0.82
14	F	51	RIGHT	IDC	2	II/III	NO	T1N0M0	IA	7.93	3.66
15	F	53	RIGHT	IDC	2.5	I/III	NO	T2N0M0	IIA	39.75	1.58
16	F	33	RIGHT	IDC	3	III/III	NO	T2N0M0	IIA	28.51	1.65
17	F	46	LEFT	IDC	1.8	III/III	NO	T1N0M0	IA	52.96	0.8
18	F	28	RIGHT	IDC	3.5	II/III	NO	T2N0M0	IIA	32.58	0.82
19	F	52	RIGHT	IDC	3.8	II/III	NO	T2N0M0	IIA	11.65	11.59
20	F	40	RIGHT	IDC	2.5	III/III	YES	T2N1M0	IIB	26.13	1.03
21	F	55	LEFT	IDC	1.2	II/III	NO	T1N0M0	IA	55.87	1.22
22	F	46	RIGHT	IDC	2	I/III	NO	T1N0M0	IA	62.16	4.5
23	F	51	LEFT	IDC	3.5	III/III	YES	T2N3M0	IIIC	15.02	2.19

Pt = patient ID; S = sex; A (y) = age in years; IDC = invasive ductal carcinoma; C Ki-67 index = cumulative Ki-67 index of patient calculated by the expert annotations in percent; C TILs score = cumulative TILs score of patient calculated by the expert annotations in percent.

**Table 2:** Statistics of the annotated cells.

Cell type	Total set (2357 IMG)		Training set (1656 IMG)		Test set (701 IMG)	
	# Cells	Avg./IMG	# Cells	Avg./IMG	# Cells	Avg./IMG
Immunopositive	50,861	21.58	35,106	21.19	15,755	22.50
Immunonegative	107,647	45.69	75,008	45.29	32,639	46.62
Lymphocyte	4490	1.90	3112	1.87	1378	1.96
Total cells	162,998	23.06	113,226	22.79	49,772	23.70

The # cells reports the number of annotated cells. avg./IMG relate average cells per image.

**Figure 1:**
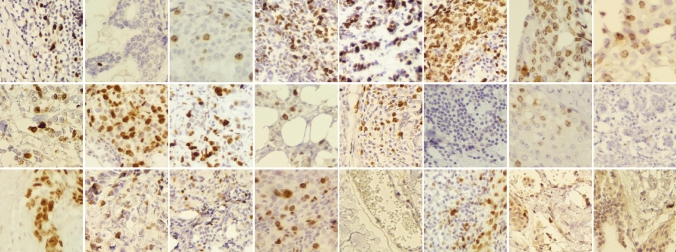
SHIDC-B-Ki-67 dataset samples.

Images were taken from slides prepared from breast mass tru-cut biopsies, which were further stained for Ki-67 by IHC method. Specific monoclonal antibodies (clone SP6) were obtained from Biocare Medical, Ca, USA. The adjuvant detection kit, named Master polymer plus detection system (peroxidase), was obtained from Master Diagnostica, Granada. Dimethylbenzene (Xylene 99.5%) obtained from Samchun Chemical Co., Ltd, South Korea, Ethanol 100% from JATA Co., Iran, and 96% from Kimia Alcohol Zanjan Co., Iran, EDTA and Tris (molecular biology grade) obtained from Pars Tous Biotechnology, Iran. Phosphate buffer saline (PBS $1{\times}$) 0.01M, pH 7.4 was prepared. At first, paraffin-embedded blocks of breast tissue were sectioned (4–5 microns) and fixed on glass slides. Prepared slides were further immunostained. Hematoxylin was used for counterstaining to perform nuclear staining and semi-quantify the extent of immunostaining that would further be evaluated. Accordingly, the expert pathologists identified the tumoral areas in each slide, by visual analysis of tissue sections under a light microscope. The final diagnosis of each case was also approved by two experienced pathologists and confirmed by some ancillary tests such as immune staining for more markers. An Olympus BX-51 system microscope with a relay lens with a magnification of $10{\times}$ coupled to OMAX microscope digital color camera A35180U3 were used to get digital images of the tumoral tissue slides. Complementary details of the camera and setup are provided in the supplementary information document. Images acquired in RGB (24-bit color depth, 8 bits per color channel) color space using $400\times$ magnification, corresponding to objective lens $40\times$. The stepwise acquisition of images is as follows: first, the pathologist identifies the tumor and defines a region of interest (ROI). In order to cover the whole ROI, several images are captured that may be overlapping. The pathologist preferentially selects images of the tumoral area, but some of the images also include transitional parts, e.g., tumoral/non-tumoral areas. A final visual (i.e., manual) inspection discards out-of-focus images. Then, stained images were labeled by expert pathologists as Ki-67 positive tumor cells, Ki-67 negative tumor cells, and tumor cells with positive infiltrating lymphocytes. Figure 1 depicts some samples of SHIDC-B-Ki-67 dataset.

**Figure 2:**
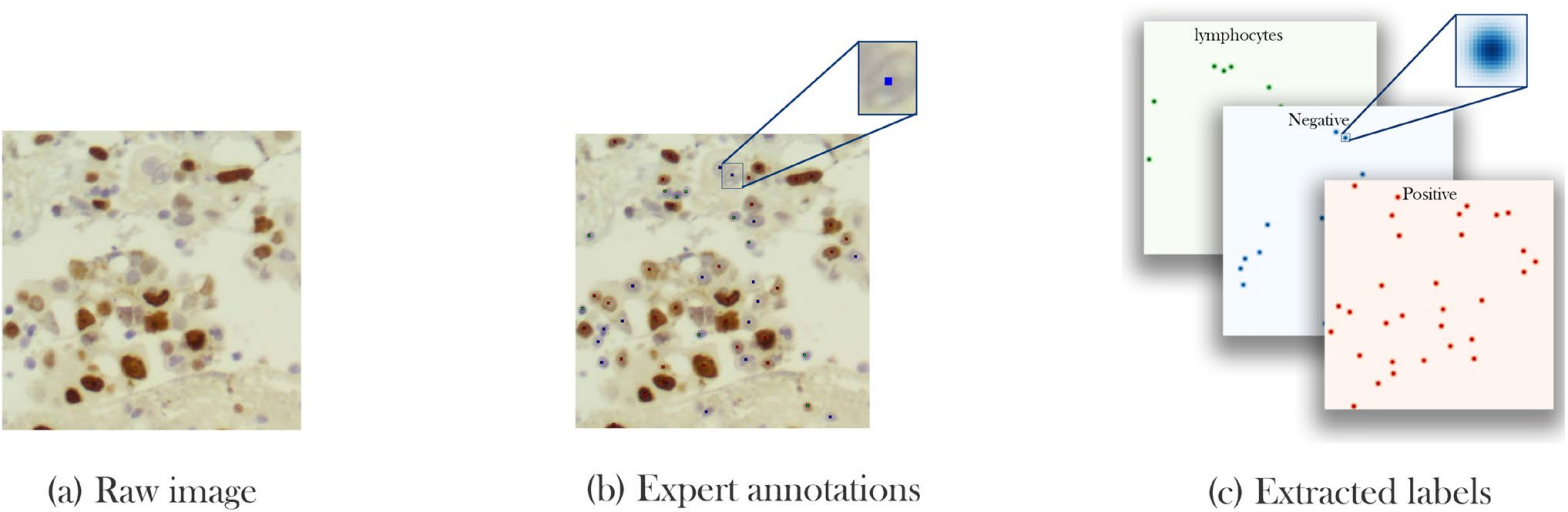
SHIDC-B-Ki-67 labeling process. (**a**) Capturing and cropping raw images, (**b**) specifying cell centers along with cell types by experts, (**c**) generating density maps from cell centers.

*Labeling* Precision and quality of expert labels play a crucial role in the correct learning process and methods’ accuracy. However, labeling real world data is a challenging and labor-intensive task. In SHIDC-B-Ki-67, each image contains 69 cells on average and a total of 162,998 cells. Manually labeling all cells, requires the time, effort and precision of experts that may be tiresome and error-prone for large numbers of samples. Another big challenge in the process is choosing a label type. In the segmentation task, labeling requires determining a class for each pixel; however, due to overlapping pixels in many cells and the infeasibility of annotating each pixel on histopathological images scale, this approach is not applicable in our case. In addition, annotations of detection tasks are usually a bounding box around the object of interest. Utilizing this type of annotation in our case where cells are small and abundant with different sizes, makes the network design procedure more complicated. To overcome this issue, cell center plus cell type are selected as the annotation. Figure 2 demonstrates labels in this study.

Although this type of annotations hastens labeling procedure, it is not without limitations. Since just one pixel is picked as the center of each cell, many pixels exist without a label. This makes the data unbalanced and, in turn, a more laborious learning process is needed. Furthermore, this labeling approach is not appropriate for most of the ordinary neural network loss functions because they cannot be a suitable representative of loss in the task. To clarify this issue, we bring an example in which a network predicts the center of a cell with either 2-pixel or 200-pixel drift compared to the center pixel picked by experts. Normal loss functions cannot discriminate between these two pixels and scores them equally. Also, experts’ annotations are error-prone that may cause the same problem. This issue can be addressed by considering an uncertainty for center pixels annotated by the experts. The uncertainty is modeled as a Gaussian distribution with the labeled pixel as the center and n-pixel variance for each cell. As a result, instead of having a 3-channel pixel as the label, a density map for each class is used. Consequently, the nature of the problem is converted into a density map estimation problem.

## Methodology

In the following sections, we explain our suggested pipeline for cell classification and detection of Ki-67 and TILs. The pipeline takes advantage of using CNN to extract features and estimate density maps from an input RGB image.

*U-Net* is one of the most commonly used architectures in biomedical image segmentation^35^ that consists of symmetric U-shaped architecture with two paths named decoder and encoder. In each layer of the decoder, an up-sampling layer increases the feature map dimension until it reaches to the input image size. U-Net is a fully convolutional model made from 19 layers. The novelty of this method is in using skip connections between corresponding encoder and decoder layers, transmitting high detail features from encoder layers to the same size layers of the decoder. This approach leads to achieving accurate location results. Similar to many studies motivated and designed based on U-Net^36,37^, we suggest PathoNet as a backend for the proposed pipeline based on U-Net architecture.

*Residual dilated inception module* Cell detection, classification, and counting in histopathological images is a specialized and error-prone task and results may face inter-individual differences due to the nature of tissues with a variety of cell types and the high possibility of overlapping cells unless pathologists are very well experienced in the field. Nevertheless, detection of the aforementioned tumor features are crucial for the accurate diagnosis of disease and a physicians’ approach to its management. This explains the need for accuracy in developing such networks, which can mostly be done by designing a deeply structured network involving plenty of parameters, yet this mostly causes vanishing gradient issues.

**Figure 3:**
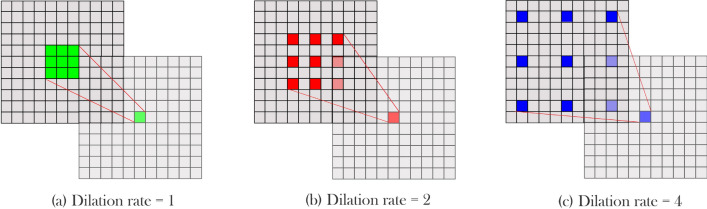
Dilated convolution visualization. Dilated convolution kernels presented with dilation rates (**a**) 1, (**b**) 2, and (**c**) 4. Increasing the dilation rate increases the kernel’s field of view.

On the other hand, cell size may vary from image to image, and since the same cell types usually sit together, picking a suitable kernel size is crucial. Szegedy et al.^38^ proposed an inception module to provide a wider field of view without having exponential parameter growth. In the inception model, instead of building a deeper network by stacking convolutional layers, parallel convolutional layers were added to make the network wider. Therefore, by increasing the number of kernels in a layer, higher accuracy can be achieved without facing the vanishing gradient problem. Also, by utilizing different kernel sizes in one module, the problem of choosing a fixed kernel size was solved, and as a result field of view increased. In the inception module proposed by Szegedy et al., three parallel kernels with 5 × 5, 3 × 3, and 1 × 1 sizes were used before using a max-pooling layer. Also, to prevent immense computation growth, input tensor channels were decreased using 1 × 1 kernels before 5 × 5 and 3 × 3 kernels. Still, their method increases network parameters, which means the network needs more data to train and is more likely to be over-fitted. To overcome this issue, Yang et al.^39^ employed dilated convolutions instead of regular convolutional layers inside the inception module. D-Dilated convolution has a D distance between each kernel element that can cover a wider region. Figure 3 shows this operator with different dilation rates. Equation (1) shows a K × K dilated convolution with step size D.

1 $$\begin{aligned} output[i] = \sum _{n=1}^K Input[i+d\cdot n]\cdot Kernel[n] \end{aligned}$$

**Figure 4:**
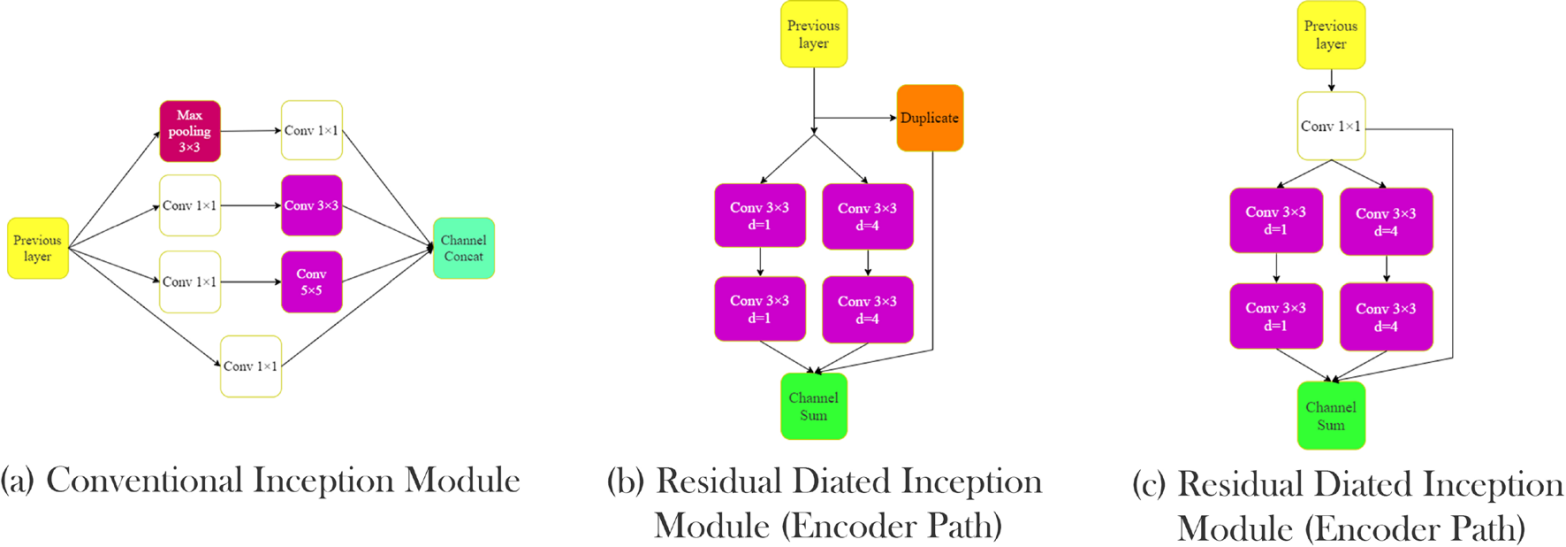
Inception modules. Comparison of conventional and proposed inception module (**a**) conventional inception module, (**b**) residual dilated inception module (encoder path), (**c**) residual dilated inception module (decoder path).

In other words, a convolution operation is a dilated convolution with a dilation rate of one. By using dilated convolution in the inception module, Yang et al.^39^ maintained the accuracy and reduced the number of model parameters. Also, instead of using multiple 1 × 1 kernels before other kernels, a 1 × 1 kernel that shares output with the next kernels was used. In the parallel convolution, outputs add together while in the inception module proposed by Szegedy et al., these outputs concat together, which consumes more memory. In a study by He et al.^40^, the authors used ResNet blocks to design deeper architectures without facing overfitting or vanishing gradient effect. In the ResNet blocks, the activation function output of layers sums up with the previous layers’ output. Motivated by the mentioned studies, in this article, we used a new inception module called residual dilated inception module (RDIM). The RDIM consists of two parallel paths where the first path has two convolution layers with kernel size 3 × 3, and the second one is built by stacking two 3 × 3 dilated convolution layers with dilation rates equal to 4. In the end, the two paths’ output sum up with the module input. However, since the number of the input channels must be the same with the output to be able to perform summation, two inception modules are used in the encoder and the decoder. In the encoder part, where the input channels are half of the output, duplicated input sums with the result of the two paths. In contrast, in the decoder, a 1 × 1 convolution with the same kernel size as the two paths applies to the input then sums with the results of parallel routes. Figure 4 shows inception and residual dilated inception module structures.

**Figure 5:**
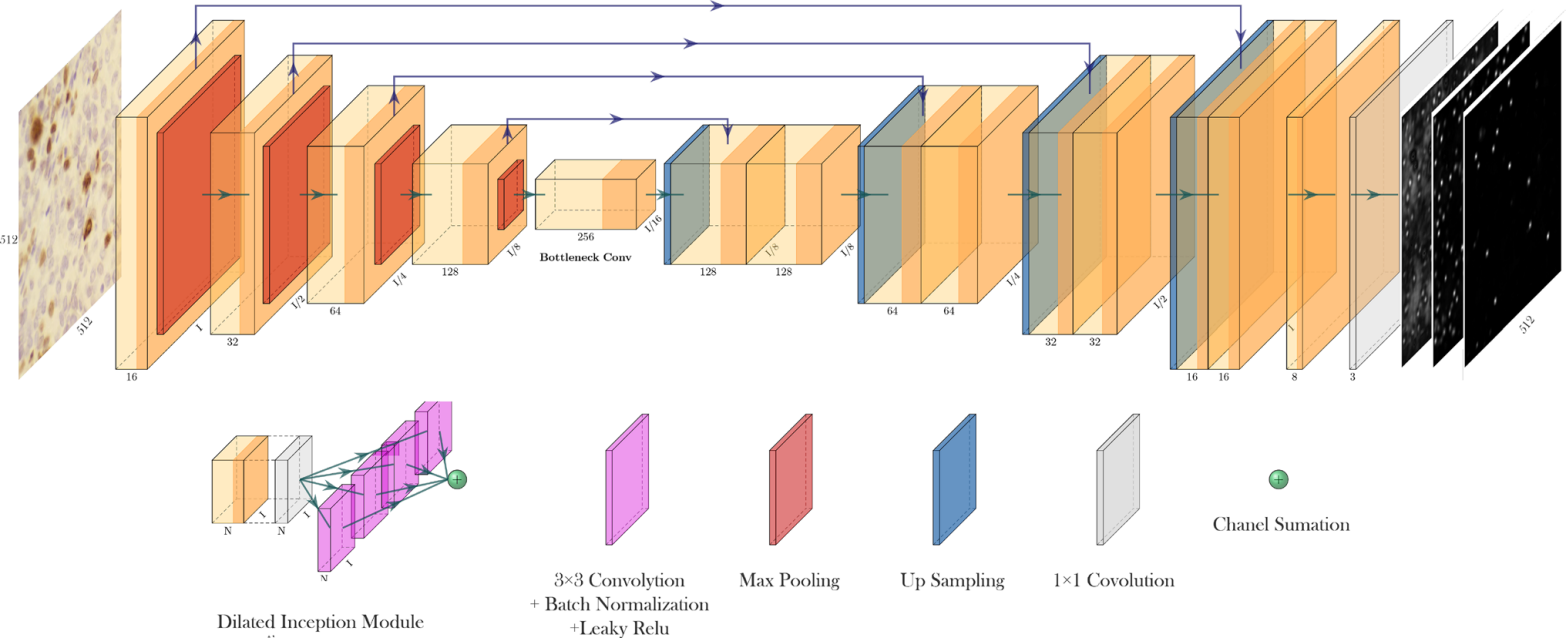
PathoNet architecture.

*PathoNet* PathoNet first extracts features from input images then predicts candidate pixels for Ki-67 immunopositive and immunonegative cells, and also lymphocytes with their corresponding density values. The proposed backend utilizes the U-Net-like backbone, where except for the first layer, convolutional layers are replaced by RDIM. In PathoNet, first, input passes through two convolutional layers. Then in the encoder, three and the decoder four RDIMs are used. In the end, a layer consists of three 1 × 1 convolution layers, and a linear activation function produces a three-channel output of the model. Figure 5 demonstrates PathoNet architecture. This network results in a three-channel, 256 by 256 matrix that each channel corresponds to the density map of Ki-67 immunopositive, immunonegative, or lymphocyte class.

**Figure 6:**
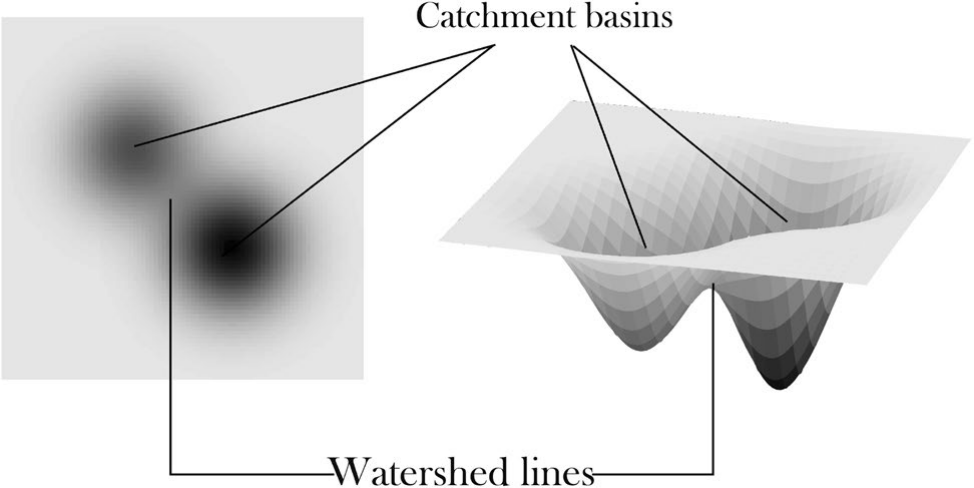
Watershed Algorithm maps grayscale images to a topographic relief space.


*Watershed* Watershed algorithm is a conventional method that is useful in medical and material science image segmentation^41^. Watershed was first introduced in 1978^42^, but over the past decades, different other versions of the method have been proposed^43^. This algorithm maps grayscale images to a topographic relief space. In a 3-dimensional relief space, each point corresponds to a pixel in the input image with height value equal to the pixel intensity. This relief space consists of different regions, namely low-lying valleys (minimums), high-altitude ridges (watershed lines), and slopes (catchment basins). These regions are demonstrated in Fig. 6. Watershed algorithm’s objective is to find catchment basins or watershed lines. Watershed is based on a simple yet useful theory. Lines connecting these points are called watershed lines that clarify the segment borders, and the holes are catchment basins or image segments. Algorithm 1 describes a simple Watershed algorithm.

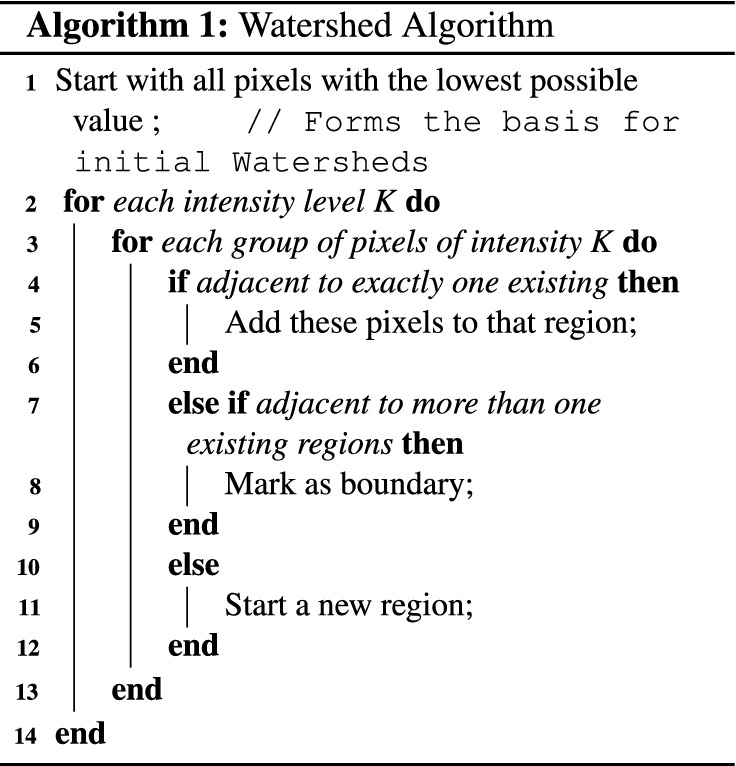


**Figure 7:**
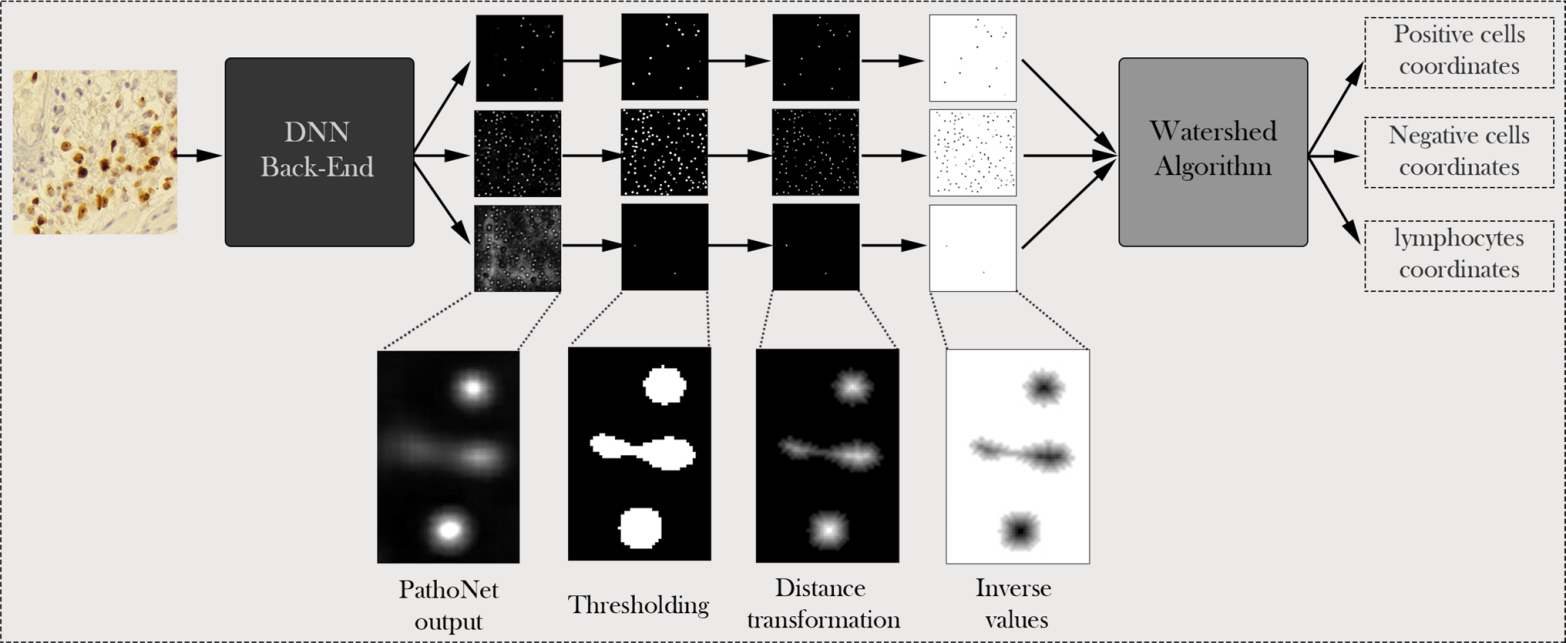
The proposed pipeline. First, a backend, which is a density map estimator based on CNNs, predicts a density map for each class. Then thresholding applies to density maps and produces binary images. Further, inversed distance transformation scores region centers with low and borders with high values. Finally, the watershed algorithm predicts cell centers, and the pipeline outputs the cell coordinates.

*Proposed pipeline* The proposed pipeline consists of three components: (1) PathoNet network, (2) post-processing, and (3) Watershed algorithm. The Watershed algorithm and post-processing components do not contain trainable elements, therefore in the training phase, we train the PathoNet component. During the test phase, PathoNet generates a 3-channel density map from an input image where each channel corresponds to the density map of a class. Since there can be multiple pixels in a small region of the map with close or equal densities, choosing a single pixel as the center is rather ambiguous. Besides, due to the presence of noise and low-density points, the amount of false-positive predictions increases. Hence, a post-processing stage is added to the pipeline. Within the post-processing stage, at first, points with less than the specified threshold are removed and points more than the threshold maps to 255. Second, distance transformation is applied on each channel, producing a grayscale image in which the value of points in continuous regions shows the distance from region borders. After applying distance transformation, areas with only one maximum point are produced. For non-circular regions or where we have overlapping cells, there might be multiple maximum points. Finally, we apply the Watershed algorithm that produces cell center coordinates to segment these regions and the overlapping cells. It is notable that because Watershed finds minimum values, an inverse operation is applied before performing the Watershed method in the third step of the post-processing phase. Figure 7 presents the proposed pipeline.

*Experimental setup* In order to obtain a balanced train and test set, 70% of each patient’s images were randomly selected for the training and 30% for the test set. The resulted splits are included in the dataset files. Then all the methods are trained on the training set, and results are reported on the test set. MSE loss function by means of the ADAM optimizer is used for training. Learning rate was empirically set to 0.0001 with a 0.1 decrease rate every ten epochs. Keras framework^44^ was also used to train the networks using two NVIDIA Geforce GTX 1060 and an Intel Core-i5 6400 processor.

## Results and discussion

To the best of our knowledge, most of the methods introduced for automated detection of Ki-67 have reported their results on datasets that are not publicly available, Therefore in this study, the authors not only presented a publicly available dataset, but also introduced a backend for a more precise estimation Ki-67 index. Our presented method overrules others also in generating pixel coordinates in addition to cell class types, as for other studies, only prediction of cell nuclei or Ki-67 score is reported^27,29^. Since no previously reported method exists that can be applied directly on our classification and detection benchmark, comparison of the currently presented method was made with state of the art methods as the backend in our pipeline and results were reported accordingly. DeepLabV3^45^ that has the best results on the VOC-PASCAL 2012^46^ was used. The last layer of DeepLabV3 was replaced with a 3-channel convolution layer that has a linear activation function and can have outputs similar to PathoNet. The DeepLab method has Mobilenet and Xeption implementations, which we have provided results for both. Similar to PathoNet, FCRN-A, and FCRN-B^47^ were designed for density estimation but at a single class. For evaluating FCRN-A and FCRN-B methods as our pipeline backend, their last layer was changed from one kernel to three kernels. Since PathoNet backbone is similar to U-Net, we trained it in the same setting as the other methods did, yet, U-Net proved to be underfitting and, therefore, could not be evaluated. Though, by following the methods presented the by Zhou et al.^48^, a modified version of U-Net with batch normalization^49^ layers of this study were evaluated. Evaluation results of the proposed pipeline with different backends is provided in Table 3.

**Table 3:** Cell detection and classification results.

Backend	Immunopositive			Immunonegative			Lymphocyte			Average		
	Prec.	Rec.	F1	Prec.	Rec.	F1	Prec.	Rec.	F1	Prec.	Rec.	F1
Modified DeepLabv3-Mobilenetv2^45^	0.8398	0.8252	0.8324	0.7201	0.7468	0.7332	0.1170	0.3471	0.2344	0.7430	0.7606	0.7508
Modified DeepLabv3-Xeption^45^	0.8366	0.8659	0.8510	0.7168	**0.8330**	0.7705	0.4227	**0.4717**	**0.4458**	0.7467	**0.8334**	0.7871
Modified FCRN-A^47^	0.8287	0.8556	0.8419	0.7190	0.7887	0.7523	0.3950	0.4101	0.4028	0.7449	0.7994	0.7710
Modified FCRN-B^47^	0.8270	**0.8726**	0.8492	0.7367	0.7908	0.7628	**0.4253**	0.7376	0.4314	0.7568	0.8069	0.7810
Modified U-Net^35^	0.8415	0.8436	0.8426	0.7351	0.7899	0.7615	0.4146	0.4630	0.4375	0.7600	0.7972	0.7783
Proposed method	**0.8436**	0.8611	**0.8523**	**0.7466**	0.8198	**0.7815**	0.3424	0.4246	0.379	**0.7766**	0.8219	**0.7928**

Measurements on the test set in terms of precision (Prec.), recall (Rec.), and F1-score (F1.) reported based on different backends. Best results in each column have been shown in bold font.

*Measurements* To evaluate our pipeline, first, we need to define true and false predictions. We count an estimation as True positive (TP) when the predicted center has a less than R pixel distance with the corresponding ground truth; otherwise, it is marked as a False positive(FP). If more than one detected center is within an R-pixel distance with the same cell type in the ground truth, estimation with lower distance counts as TP and otherwise as FP. Finally, cells are defined in the ground truth, but without any prediction for False Negatives (FN). With the given definitions, the precision and recall formulas are shown in Eq. (2).

2 $$\begin{aligned} Precision = \frac{TP}{TP+FP} \quad Recall = \frac{TP}{TP+FN} \end{aligned}$$

A model with high recall and low precision rates detects most of the pixels as cell centers, while most of them are FP. On the other hand, low recall and high precision rates in a model bring about the detection of few cells, while most detected cells are TP. None of the mentioned cases is useful; therefore, our goal is to develop a model that holds a trade-off between precision and recall. F1 score or harmonic mean is an appropriate measure that can be used for this evaluation that can be calculated using Eq. (3).

3 $$\begin{aligned} F1-score = 2 \cdot \frac{Precision \cdot Recall}{ Precision + Recall} \end{aligned}$$

Considering the importance of precise estimation of both markers, we also evaluated the introduced pipeline with using different backends and the performance of the proposed model in terms of TILs and Ki-67 index calculation was evaluated using RMSE.

4 $$\begin{aligned}&Ki-67-score=\frac{Immunopositive}{Immunopositive+Immunonegative} \end{aligned}$$

5 $$\begin{aligned}&TIL-score = \frac{Lymphocyte}{Lymphocyte + Immunopositive+Immunonegative} \end{aligned}$$

**Figure 8:**
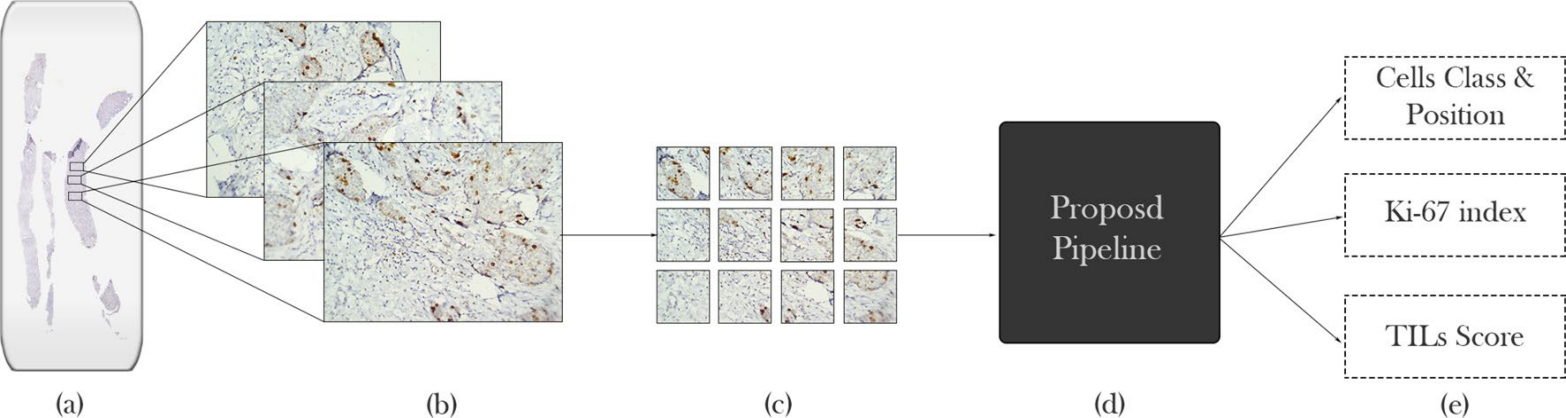
From whole mount slide image preparation to Ki-67 and TILs estimation. The process starts by taking pictures (**b**) from a patient’s tumor section (**a**) and cropping them into 256 × 256 pixels sub-images. Next, each cropped image (**c**) is fed to the proposed pipeline (**d**), resulting in predicted cell centers, corresponding class labels (**e**). Then, the Ki-67 index and TILs score can easily be calculated. Figure 10 depicts a prediction sample image with $4912\times 3684$ pixels size.

Also, since each raw image is cropped into smaller ones, we grouped all images that belonged to a patient into one group and classified the Ki-67 ane TILs estimation into different cut-off categories that have been presented in previous studies (Fig. 8). This aggregated-image classification results are presented under TILs and Ki-67 cut-off accuracy column of Table 4. As suggested by Saha et al.^26^, cases with Ki-67 scores below 16 percent are accounted as less proliferative, between 16 and 30 as having average proliferation rate, and higher than 30 as highly proliferative. Also, based on TILs score, the cut-off ranges presented in literature is between 0 and 10, between 11 and 39, and higher than 40%^8^. Table 4 compares RMSE and accuracy of different backends based on the proposed pipeline for Ki-67 and TILs scoring.

**Table 4:** RMSE and aggregated cut-off accuracy.

Backend	Ki-67 index (RMSE)	TILs score (RMSE)	Ki-67 cut-off accuracy	TILs cut-off accuracy
Modified DeepLabv3-Mobilenetv2^45^	0.05123	0.05568	0.9565	0.8260
Modified DeepLabv3-Xeption^45^	0.06341	0.01639	0.9130	0.9565
Modified FCRN-A^47^	0.05836	0.01580	0.9565	1.0
Modified FCRN-B^47^	0.06285	0.01674	0.9565	1.0
Modified U-Net^35^	0.05206	0.01497	0.9556	0.9565
Ours(PathoNet)	0.04803	0.02530	0.9565	0.9565

Here, Ki-67 index and TILs score estimation were evaluated in terms of root mean squared error (RMSE) and classification accuracy of cut-offs presented for Ki-67 and TILs using the proposed pipeline on the test set.

*Quantitative results* As shown in Table 3, DeepLabv3-Xeption performed better in Ki-67 immunopositive cell detection. However, our introduced model outperforms the others in the detection of Ki-67 immunonegative cells in terms of precision and harmonic mean (F1 score). The suggested pipeline using FCRN-B has better precision and harmonic mean in the lymphocyte class of cells. In contrast, our model has a better recall rate, meaning that the pipeline using PathoNet could detect more labeled lymphocytes than the other methods. The proposed backend performed better in terms of precision by having the lowest FP and outperforming the others in terms of overall F1. Although DeepLabv3-Xception is very close to ours in the overall F1, as shown in Table 5, it has 12 times more parameters compared to the proposed method. So, not only DeepLabv3-Xception needs more computation resources for training, but also, the proposed method can be processed faster and provides higher FPS while maintaining a better F1 score than DeepLabv3-Xception.

**Table 5:** Inference speed and model parameters.

Backend	# Parameters	FPS	Avg. F1
Modified DeepLabv3-Mobilenetv2^45^	3,236,907	20.52	0.7508
Modified DeepLabv3-Xeption^45^	41,253,587	8.76	0.7871
Modified FCRN-A^47^	2,142,019	22.03	0.7710
Modified FCRN-B^47^	1,365,888	14.6	0.7810
Modified U-Net^35^	31,036,323	12.34	0.7783
Ours (PathoNet)	3,142,208	14.86	0.7928

Run-time in terms of frame per seconds and models’ parameter counts of different backends used in the proposed pipeline.

**Figure 9:**
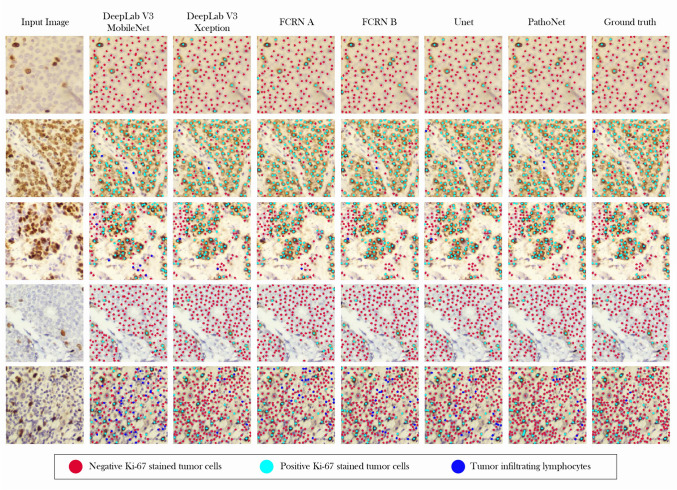
Qualitative results. Samples from SHIDC-B-Ki-67 test set. Points in red show negative Ki-67 stained tumor cells. Blue points depict positive Ki-67 stained tumor cells and dots with cyan color, show tumor infiltrating lymphocytes. In this image, ground truth column is expert annotation.

**Figure 10:**
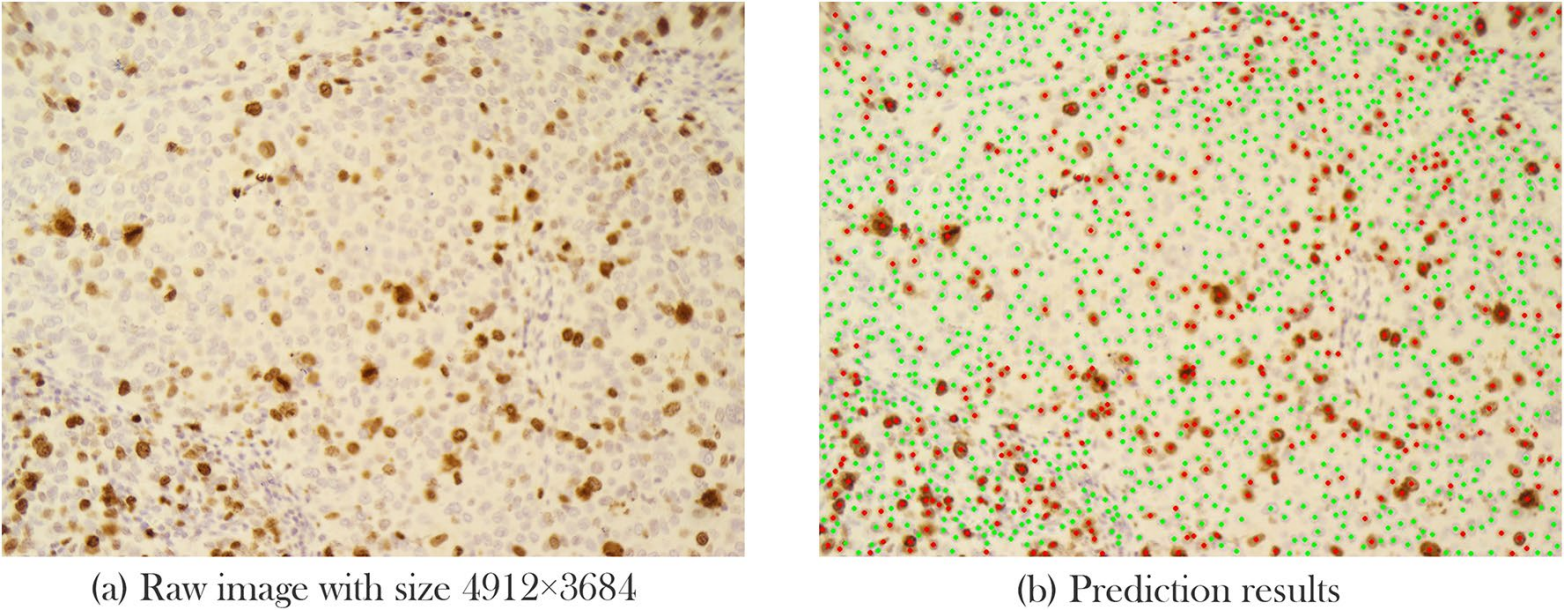
Aggregated sub-image qualitative results. Input raw image is cropped into smaller sub-images and, after prediction, aggregates to build the input image. This sample is visualized from the test set.

*Qualitative results* We elaborated the qualitative results on the SHIDC-BC-Ki-67 test set in Fig. 9. We have compared our proposed pipeline’s detection and classification results with different backends in addition to the ground truth and the input images that have been presented. The proposed pipeline leads to the detection of highly overlapped cells with different colors, sizes, and lighting conditions as shown in Fig. 9. Compared to the required clinical ROI, $255 \times 255$ images are small. Considering the quantitative aggregated scores of patients presented in Table 4, by following the process in Fig. 8, qualitative results of aggregated sub-images of a patient is presented in Fig. 10.

*Limitations* Variations of tumoral cell size and color from patient to patient complicates labeling and annotation of cells. Also using only Ki-67 marked images for TILs scoring leads to masking of Ki-67 positive TILs. This issue could be resolved by dual IHC staining which was unavailable at our center. However, we believe that the powerful deep model feature extractors presented, have shown promising results in overcoming this issue. We should mention that the recommended staining for evaluation of TILs is Hematoxylin and Eosin (H&E), but since during IHC staining, a counter staining process is performed using Hematoxylin, we expected the lymphocytes’ nuclei be stained sufficiently as has been recommended by guidelines.

## Conclusion

In this article we introduced a new benchmark for cell detection, classification , proliferation index estimation, and TILs scoring using histopathological images. We further proposed a new pipeline to be used in cell detection and classification that can utilize different deep models for feature extraction. We evaluated this pipeline on immunonegative, immunopositive, and TILs cell detection on Ki-67 stained images. Also, we suggested a residual inception and designed a new light-weight backbone called PathoNet that achieved state of the art results on the proposed dataset. The suggested pipeline showed high accuracy in Ki-67 and TILs cut-off classification compared with all backends. Finally, we showed that, designing PathoNet using RDIM provides high accuracy while slightly increasing model parameters.

## Supplementary Information

Supplementary Informations.

## Data Availability

All analysis results and ablations withing this study are included is this article. In addition, dataset is publicly available and can be accessed from http://www.shiraz-hidc.com.

## Code availability

Codes and scripts to replicate the results is provided on the related repository https://github.com/SHIDCenter/PathoNet.

## References

[1] Gerdes J, Schwab U, Lemke H, Stein H (1983). Production of a mouse monoclonal antibody reactive with a human nuclear antigen associated with cell proliferation. Int. J. Cancer.

[2] Gerdes J (1984). Cell cycle analysis of a cell proliferation-associated human nuclear antigen defined by the monoclonal antibody ki-67. J. Immunol..

[3] Lopez F (1991). Modalities of synthesis of ki67 antigen during the stimulation of lymphocytes. Cytom. J. Int. Soc. Anal. Cytol..

[4] Dowsett M, Dunbier AK (2008). Emerging biomarkers and new understanding of traditional markers in personalized therapy for breast cancer. Clin. Cancer Res..

[5] Jones RL (2009). The prognostic significance of ki67 before and after neoadjuvant chemotherapy in breast cancer. Breast Cancer Res. Treat..

[6] Taneja P (2010). Classical and novel prognostic markers for breast cancer and their clinical significance. Clin. Med. Insights Oncol..

[7] Denkert C (2010). Tumor-associated lymphocytes as an independent predictor of response to neoadjuvant chemotherapy in breast cancer. J. Clin. Oncol..

[8] Denkert C (2018). Tumour-infiltrating lymphocytes and prognosis in different subtypes of breast cancer: a pooled analysis of 3771 patients treated with neoadjuvant therapy. Lancet Oncol..

[9] Mao Y (2014). The value of tumor infiltrating lymphocytes (TILs) for predicting response to neoadjuvant chemotherapy in breast cancer: a systematic review and meta-analysis. PLoS ONE.

[10] Mao Y (2016). The prognostic value of tumor-infiltrating lymphocytes in breast cancer: a systematic review and meta-analysis. PLoS ONE.

[11] Urruticoechea A, Smith IE, Dowsett M (2005). Proliferation marker ki-67 in early breast cancer. J. Clin. Oncol..

[12] Dowsett M (2011). Assessment of ki67 in breast cancer: recommendations from the international ki67 in breast cancer working group. J. Natl. Cancer Inst..

[13] Kononenko I, Bratko I, Kukar M (1997). Application of machine learning to medical diagnosis. Mach. Learn. Data Min. Methods Appl..

[14] Soans, N., Asali, E., Hong, Y. & Doshi, P. Sa-Net: robust state-action recognition for learning from observations. In *IEEE International Conference on Robotics and Automation (ICRA)*, 2153–2159 (2020).

[15] Haskins G, Kruger U, Yan P (2020). Deep learning in medical image registration: a survey. Mach. Vis. Appl..

[16] Hafiz, A. M. & Bhat, G. M. A survey of deep learning techniques for medical diagnosis. In Tuba, M., Akashe, S. & Joshi, A. (eds) *Information and Communication Technology for Sustainable Development*, 161–170 (Springer, 2020).

[17] Krizhevsky, A., Sutskever, I. & Hinton, G. E. Imagenet classification with deep convolutional neural networks. *Advances in Neural Information Processing Systems*, 1097–1105 (2012).

[18] Xing F, Su H, Neltner J, Yang L (2013). Automatic ki-67 counting using robust cell detection and online dictionary learning. IEEE Trans. Biomed. Eng..

[19] Swiderska, Z., Markiewicz, T., Grala, B. & Slodkowska, J. Hot-spot selection and evaluation methods for whole slice images of meningiomas and oligodendrogliomas. In *2015 37th Annual International Conference of the IEEE Engineering in Medicine and Biology Society (EMBC)*, 6252–6256 (IEEE, 2015). 10.1109/EMBC.2015.7319821 26737721

[20] Shi P (2016). Automated ki-67 quantification of immunohistochemical staining image of human nasopharyngeal carcinoma xenografts. Sci. Rep..

[21] Geread RS (2019). Ihc colour histograms for unsupervised ki67 proliferation index calculation. Front. Bioeng. Biotechnol..

[22] Xu, Y. *et al.* Deep learning of feature representation with multiple instance learning for medical image analysis. In *2014 IEEE International Conference on Acoustics, Speech and Signal Processing (ICASSP)*, 1626–1630 (IEEE, 2014).

[23] Weidi, X., Noble, J. A. & Zisserman, A. Microscopy cell counting with fully convolutional regression networks. In *1st Deep Learning Workshop, Medical Image Computing and Computer-Assisted Intervention (MICCAI)* (2015).

[24] Paul Cohen, J., Boucher, G., Glastonbury, C. A., Lo, H. Z. & Bengio, Y. Count-ception: counting by fully convolutional redundant counting. In *Proceedings of the IEEE International Conference on Computer Vision*, 18–26 (2017).

[25] Spanhol, F. A., Oliveira, L. S., Cavalin, P. R., Petitjean, C. & Heutte, L. Deep features for breast cancer histopathological image classification. In *2017 IEEE International Conference on Systems, Man, and Cybernetics (SMC)*, 1868–1873 (IEEE, 2017).

[26] Saha M, Chakraborty C, Arun I, Ahmed R, Chatterjee S (2017). An advanced deep learning approach for ki-67 stained hotspot detection and proliferation rate scoring for prognostic evaluation of breast cancer. Sci. Rep..

[27] Zhang R (2018). Tumor cell identification in ki-67 images on deep learning. Mol. Cell. Biomech..

[28] Sornapudi S (2018). Deep learning nuclei detection in digitized histology images by superpixels. J. Pathol. Inform..

[29] Jiang Y, Chen L, Zhang H, Xiao X (2019). Breast cancer histopathological image classification using convolutional neural networks with small SE-ResNet module. PLoS ONE.

[30] Liu, Q., Junker, A., Murakami, K. & Hu, P. A novel convolutional regression network for cell counting. In *2019 IEEE 7th International Conference on Bioinformatics and Computational Biology (ICBCB)*, 44–49 (IEEE, 2019).

[31] Spanhol, F. A., Oliveira, L. S., Petitjean, C. & Heutte, L. Breast cancer histopathological image classification using convolutional neural networks. In *2016 International Joint Conference on Neural Networks (IJCNN)*, 2560–2567 (IEEE, 2016).

[32] Lempitsky, V. & Zisserman, A. Learning to count objects in images. In *Advances in Neural Information Processing Systems*, 23, 1324–1332 (2010).

[33] Kainz, P., Urschler, M., Schulter, S., Wohlhart, P. & Lepetit, V. You should use regression to detect cells. In *International Conference on Medical Image Computing and Computer-Assisted Intervention*, 276–283 (Springer, 2015).

[34] Marsden, M., McGuinness, K., Little, S., Keogh, C. E. & O’Connor, N. E. People, penguins and petri dishes: adapting object counting models to new visual domains and object types without forgetting. In *Proceedings of the IEEE Conference on Computer Vision and Pattern Recognition*, 8070–8079 (2018).

[35] Ronneberger, O., Fischer, P. & Brox, T. U-net: convolutional networks for biomedical image segmentation. In *International Conference on Medical Image Computing and Computer-assisted Intervention*, 234–241 (Springer, 2015).

[36] Myronenko, A. 3D MRI brain tumor segmentation using autoencoder regularization. In *International MICCAI Brainlesion Workshop*, 311–320 (Springer, 2018).

[37] Dolz, J., Desrosiers, C. & Ayed, I. B. IVD-Net: Intervertebral disc localization and segmentation in MRI with a multi-modal UNet. In *International Workshop and Challenge on Computational Methods and Clinical Applications for Spine Imaging*, 130–143 (Springer, 2018).

[38] Szegedy, C. *et al.* Going deeper with convolutions. In *Proceedings of the IEEE Conference on Computer Vision and Pattern Recognition*, 1–9 (2015).

[39] Yang, S., Lin, G., Jiang, Q. & Lin, W. A dilated inception network for visual saliency prediction (2019). arXiv:1904.03571.

[40] He, K., Zhang, X., Ren, S. & Sun, J. Deep residual learning for image recognition. In *Proceedings of the IEEE Conference on Computer Vision and Pattern Recognition*, 770–778 (2016).

[41] Atta-Fosu T (2016). 3D clumped cell segmentation using curvature based seeded watershed. J. Imaging.

[42] Lantuéjoul, C. La squelettisation et son application aux mesures topologiques des mosaiques polycristallines (1978).

[43] Kornilov AS, Safonov IV (2018). An overview of watershed algorithm implementations in open source libraries. J. Imaging.

[44] Chollet, F. *et al.* Keras. https://keras.io/. Accessed 30 May 2019 (2019).

[45] Chen, L.-C., Papandreou, G., Schroff, F. & Adam, H. Rethinking atrous convolution for semantic image segmentation. *arXiv preprint* arXiv:1706.05587 (2017).

[46] Everingham M, Van Gool L, Williams CKI, Winn J, Zisserman A (2010). The pascal visual object classes (VOC) challenge. Int. J. Comput. Vis..

[47] Xie W, Noble JA, Zisserman A (2018). Microscopy cell counting and detection with fully convolutional regression networks. Comput. Methods Biomech. Biomed. Eng. Imaging Vis..

[48] Zhou T, Ruan S, Canu S (2019). A review: Deep learning for medical image segmentation using multi-modality fusion. Array.

[49] Ioffe, S. & Szegedy, C. Batch normalization: accelerating deep network training by reducing internal covariate shift. *arXiv preprint* arXiv:1502.03167 (2015).

