# Supplementary material for: PathoNet introduced as a deep neural network backend for evaluation of Ki-67 and tumor-infiltrating lymphocytes in breast cancer
Source: Sci Rep. 2021 Apr 19;11:8489. doi: 10.1038/s41598-021-86912-w (PMC8055887; doi:10.1038/s41598-021-86912-w)
Supplement: Supplementary file 1 — Supplementary Informations. [file 41598_2021_86912_MOESM1_ESM.pdf]

PathoNet introduced as a deep neural network backend for evaluation of Ki-67 and tumor-infiltrating lymphocytes in breast cancer

Farzin Negahbani[1,7], Rasool Sabzi[1], Bita Pakniyat Jahromi[2], Fateme Movahedi[3], Mahsa Kohandel Shirazi[2], Shayan Majidi[3], Dena Firouzabadi[4], and Amirreza Dehghanian[5,6,*]

[1]Department of Computer Science and Engineering, Shiraz University, Shiraz, Iran.
[2]Department of Pathology, Shiraz University of medical Science, Shiraz, Iran.
[3]Student Research Committee, Shiraz University of medical Science, Shiraz, Iran.
[4]Department of Clinical Pharmacy, Shiraz University of Medical Sciences, Shiraz, Iran
[5]Molecular Pathology and Cytogenetics division, Department of Pathology, Shiraz University of Medical Sciences, Shiraz, Iran.
[6]Trauma Research Center, Shiraz University of Medical Sciences, Shiraz, Iran.
[7]Department of Computer Science and Engineering, Koc University, Istanbul, Turkey.
[*]adehghan@sums.ac.ir

Appendix A

This document contains complementary data to replicate our results. In the following sections, details about data preprocessing, parameters, threshold tuning procedure, web-based labeling tool, and imaging setup information are provided.

A.1 Imaging Setup

The camera sensor used for imaging has the following specifications: Aptina 1/2.3 inch color CMOS with a resolution equal to 4912x3684 pixels (18 megapixels) with pixel size about 1.25um x 1.25um; Complementary details for the mentioned camera are as follows: SNR: 36.3 dB; Dynamic Range: 65.8 dB; Frame speed: 5.6 FPS at 4912x3684, 18.1 FPS at 2456x1842, 32.2 FPS at 1228x922.

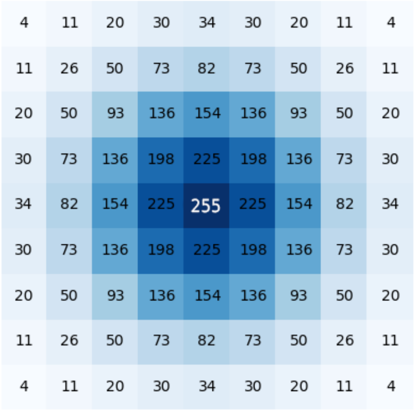

Figure 1. Gaussian Distribution as Label.

Method	Label Type	F1 Score			
		IHC Positive	IHC Negative	TILs	Total
Proposed Method (PathoNet)	Center pixel	0.814	0.713	**0.401**	0.736
	Gaussian (center 255)	**0.852**	**0.781**	0.379	**0.792**
Modified U-Net	Center pixel	0.815	0.745	0.412	0.758
	Gaussian (center 255)	**0.843**	**0.762**	**0.438**	**0.778**

Table 1. **Effect of Label Type Ablations.** Results reported on the test set. Center pixel shows the case only one pixel picked as the GT. Gaussian (center 255) illustrates when Gaussian distribution is employed as the GT.

A.2 Data Label Representation

The motivation behind using a distribution over the center pixel as ground truth (GT) is to prevent the data from becoming unbalanced. This motivation is further investigated in Table. 1 that shows the effect of label type on the models' performance. To obtain GT, after determining a center pixel by experts, a Gaussian distribution fits on the center with a variance of 9 pixels and the maximum value equal to 255. Figure. 1 represents discrete Gaussian distribution used in this study.

A.3 SHIDC-Lab Software

In this study, experts used SHIDC-Lab to annotate the images. SHIDC-Lab is designed to provide flexibility, speed, easy monitoring capability, and revision ability that can be run on any device with a web browser so that experts can perform labeling in their spare time. In SHIDC-Lab software, the process starts by uploading images to the server and defining annotation classes, corresponding signs, and colors. In our case, immunopositive, immunonegative, and TILs types are defined. Afterward, the head expert assigns images to each expert to perform labeling. Finally, head-experts revise annotations and mark images as the accepted version or re-assign them for another round of labeling.

A.4 Pre-processing and training

In order to prepare data, we first crop the 4912×3684 image and its corresponding label to non-overlapping 1228×1228 regions. Next, cropped images are resized to 256×256 pixels. Then the dataset, is split into two parts; 0.7 of the data for training and 0.3 for the test. Further, data augmentation is performed by flipping images with respect to x and y coordinates as well as 90, 180, and 270 degrees rotations. The final data after performing data augmentation has 8,280 images and 566,130 cells.

In training PathoNet, the simple MSE loss function by means of the ADAM optimizer is used. The learning rate is set to 0.0001 and decreases with a 0.1 rate every ten epochs. Keras framework was used in this study to train the network using two NVIDIA Geforce GTX 1060 and an Intel Core-i5 6400 processor.

A.5 Threshold Tuning

As explained in the Methodology section, to extract cell center pixels from the network's density map, thresholding is applied. To tune this threshold for each model, all values from 0 to 255 with a step size of 5 have been evaluated in terms of the F1-score of the cell predictions and the best threshold picked for each model. Table. 2 shows the tuned thresholds in this study.

Model	Cell Type		
	Positive	Negative	TIL
Modified DeepLabv3-Mobilenetv2	25	25	5
Modified DeepLabv3-Xeption	55	40	45
Modified FCRN-A	70	65	40
Modified FCRN-B	60	65	40
Modified Unet	40	30	45
Ours (PathoNet)	60	55	40

Table 2. Threshold values used in this study. Tuned values are provided to help replicate the same results as this study.

A.6 Backends Evaluation on Different sets.

In Table. 3, the proposed framework with different backends trained on the train set and evaluated on the train set, test set, and train+test set. Our method shows to perform better on the test set compared to other methods. U-Net obtains higher scores on

Backend	Testing Data	Immunopositive	Immunonegative	Lymphocytes	Average	Model Params
Modified DeepLabv3-Mobilenetv2	Test	0.832	0.733	0.234	0.750	3,236,907
	Train	0.849	0.758	0.227	0.771	
	Train+Test	0.844	0.749	0.229	0.764	
Modified DeepLabv3-Xeption	Test	0.851	0.770	0.445	0.787	41,253,587
	Train	0.875	0.819	0.492	0.827	
	Train+Test	0.867	0.803	0.476	0.814	
Modified FCRN-A	Test	0.841	0.752	0.402	0.771	2,142,019
	Train	0.875	0.799	0.451	0.813	
	Train+Test	0.864	0.784	0.435	0.799	
Modified FCRN-B	Test	0.849	0.762	0.431	0.781	1,365,888
	Train	0.867	0.789	0.451	0.804	
	Train+Test	0.861	0.781	0.445	0.797	
Modified Unet	Test	0.842	0.761	0.437	0.778	31,036,323
	Train	0.924	0.891	0.540	0.891	
	Train+Test	0.898	0.849	0.510	0.855	
Ours (PathoNet)	Test	0.852	0.781	0.379	0.792	3,142,208
	Train	0.876	0.829	0.466	0.833	
	Train+Test	0.868	0.813	0.440	0.820	

Table 3. **F1 Scores on Different Sets.** All backends trained using the train set and further tested on the test, train, and train+test sets.

the train and test+train sets but performs poorly on the test set. The results indicate that U-Net couldn't generalize well on the task that may come from 1) the high number of parameters 2) simple architecture compared to the rest of the models. Results of DeepLabv3-Xeption puts the first hypothesis aside because DeepLabv3-Xeption has approximately 32% more parameters than the U-Net. We believe that the U-Net structure is a powerful yet simple structure that needs modifications to be utilized in more complex tasks. In this study, by designing a U-Net architecture with residual and dilated inception modules, we demonstrated a candidate for such a method with fewer parameters yet capable of generalizing complex tasks.

A.7 Watershed Algorithm Parameters.

In this study, the implementation of the Watershed algorithm from the Skimage library was used. To improve the Watershed algorithm's accuracy, the maximum points of the density map passed to the algorithm as the initial minimum points. As another parameter to this algorithm, the minimum distance between two maximum points is picked as 5.
